# Hydrophobic remodeling of the SARS-CoV-2 RBD large loop attenuates ACE2 engagement and viral fitness

**DOI:** 10.1371/journal.ppat.1014742

**Published:** 2026-10-26

**Authors:** Aojie Wang, Huimin Guo, Haonan Yang, Haojin Chen, Anna Imojara, Bin Ju, Zheng Zhang, Renhong Yan

**Affiliations:** 1 Department of Biochemistry, Key University Laboratory of Metabolism and Health of Guangdong, SUSTech Homeostatic Medicine Institute, School of Medicine, Institute for Biological Electron Microscopy, Southern University of Science and Technology, Shenzhen, Guangdong, China; 2 Key Laboratory of Zoonoses, Ministry of Agriculture and Rural Affairs, South China Agricultural University, Guangzhou, Guangdong, China; 3 Department of Biochemistry, Institute for Hepatology, National Clinical Research Center for Infectious and Communicable Diseases, Shenzhen Third People’s Hospital, the Second Affiliated Hospital, School of Medicine, Southern University of Science and Technology, Shenzhen, Guangdong, China; 4 Institute of Human Immunology, Shenzhen Medical Academy of Research and Translation (SMART), Shenzhen, Guangdong, China; 5 Center for Artificial Intelligence Driven Medical Research, Shenzhen Institutes of Advanced Technology, Chinese Academy of Sciences, Shenzhen, China; Institut de Biologia Molecular de Barcelona IBMB-CSIC, SPAIN

## Abstract

The SARS-CoV-2 Omicron subvariant JN.4 harbors a unique A475V substitution within the receptor binding domain (RBD) but exhibits limited expansion compared to its parallel descendant JN.1. Here, we investigate how hydrophobic remodeling within RBD large loop (residues 475–490) modulates receptor engagement and spike function. Cryo-electron microscopy reveals that A475V induces local remodeling of the receptor-binding motif (RBM) and alters the RBD–ACE2 interaction geometry. Local refinement revealed that the bulky valine substitution disrupts the local interaction network surrounding residue 475, resulting in a 3.5-fold reduction in ACE2-binding affinity compared with BA.2.86. In contrast to the L455S substitution in JN.1, which primarily alters the receptor-binding interface, A475V within the flexible large loop influences both local loop conformation and global spike conformational properties. Systematic mutagenesis identifies A475 and F486 as functionally constrained hydrophobic residues that preserve the structural framework of ACE2 engagement despite extensive sequence variation within the surrounding loop. Together with molecular dynamics simulations and functional assays, our findings establish the RBD 475–490 large loop as a structurally plastic yet functionally constrained element that balances receptor recognition, conformational stability, and antigenic diversification. These results provide a mechanistic framework for understanding region-specific evolutionary constraints that shape SARS-CoV-2 RBD adaptation.

## Author summary

SARS-CoV-2 continues to modify its spike protein in order to enter human cells efficiently, evade antibodies, and maintain structural stability. Most previous work has emphasized mutations that alter charged or polar interactions at the interface between the receptor binding domain and ACE2. In this study we instead focus on a subtle hydrophobic change in a flexible loop of the receptor binding domain, an alanine to valine substitution at position 475 in the Omicron subvariant JN.4, and ask how it shapes viral properties. Using cryo-EM, quantitative binding measurements, molecular dynamics simulations, and cell-based infection and fusion assays, we find that this single hydrophobic substitution does more than weaken a local contact. It reconfigures the conformation of a mobile large loop spanning residues 475–490, changes the tilt and occupancy of ACE2 on the spike trimer, and ultimately reduces ACE2 binding, membrane fusion, and pseudovirus infectivity. By comparing related variants and a panel of point mutants, we identify positions A475 and F486 as hydrophobic anchor residues that support a flexible yet structurally constrained framework for ACE2 recognition. Our work illustrates how small hydrophobic changes in flexible structural elements can impose measurable fitness costs, providing a possible structural explanation for the limited expansion of some Omicron descendants, including JN.4.

## Introduction

SARS-CoV-2 is a positive-sense single-stranded RNA virus that continues to diversify despite encoding a proofreading exoribonuclease, allowing the virus to accumulate substantial genetic diversity during sustained global transmission [1]. As population-level immunity became widespread through vaccination and repeated infection, viral evolution shifted from optimizing intrinsic transmissibility toward refining immune escape while maintaining sufficient receptor engagement [2–5]. This evolutionary transition underlies the emergence and long-term dominance of Omicron sublineages, from early BA.1 to recent variants such as XEC, XFG, NB.1.8.1, and LP.8.1 [6–8].

The spike glycoprotein serves as the primary evolutionary hotspot, mediating receptor recognition and harboring neutralizing epitopes [2,9–16]. Within the RBD-ACE2 interface, structural and evolutionary studies have predominantly focused on charged or polar substitutions that directly rewire hydrogen bonds and salt bridges [17–20]. These substitutions can enhance viral traits, including transmissibility (N501Y) [21], host adaptability (Q493R) [22,23], or antiviral resistance (N439K, L452Q, Y453F, T478K, E484K, Q493R, G496S, and Y505H) [24–26].

However, hydrophobic residues play equally critical yet mechanistically distinct roles in stabilizing ACE2 engagement through van der Waals interactions and conformational anchoring [27,28]. Distinct evolutionary outcomes emerge depending on whether hydrophobic substitutions occur in structurally constrained versus flexible regions. In rigid interface cores, such as the “middle region” surrounding residue 455, hydrophobic packing is essential for stability: the Delta variant’s L452R enhanced packing with F490 and L492 to increase transmissibility [29,30]. Whereas JN.1 L455S mutation destabilizes the “middle” region of the RBD–ACE2 interface, reducing ACE2 binding and viral infectivity [31,32]. Conversely, in flexible regions, even subtle hydrophobic changes can propagate long-range conformational shifts, as exemplified by BA.2.86 F486P mutation, which remodels the RBD-ACE2 binding mode [32–34].

A particularly understudied structural element in this regard is the large receptor-contacting loop spanning residues 475–490 [32]. Unlike the rigid middle region, this flexible segment exhibits pronounced conformational mobility, directly engages ACE2, overlaps with multiple neutralizing antibody epitopes, and constitutes a recurrent mutational hotspot [35]. Despite extensive studies of RBD evolution, the functional consequences of mutations within flexible loop regions remain incompletely understood. Such regions may tolerate sequence variation through local conformational remodeling, but how hydrophobic substitutions reshape receptor-binding geometry and functional stability remains unclear.

The JN.4 subvariant presents a unique opportunity to dissect this region-specific plasticity, which briefly appeared during the JN.1 pandemic at the end of 2023 (S1A-B Fig). As a parallel descendant of BA.2.86 alongside the globally dominant JN.1, JN.4 harbors the defining A475V substitution within this large loop, a nominally conservative change replacing a small alanine with bulkier valine [36]. Apart from JN.4, A475V has been detected in various variants of the SARS-CoV-2, such as JD.1.1, JK.1, etc., and has persisted along with the ongoing spread of the novel coronavirus (S1C Fig). Unlike JN.1 L455S mutation, which disrupts the rigid middle region, A475V targets the dynamic loop region [37], offering a natural experiment to compare how hydrophobic versus polar substitutions at distinct RBD interfaces differentially impact ACE2 engagement and viral fitness.

Here, we integrate Cryo-EM, biophysical binding measurements and functional assays to elucidate how hydrophobic remodeling within the RBD 475–490 large loop regulates spike function. We demonstrate that the JN.4 A475V substitution reshapes the receptor-binding interface and attenuates ACE2 engagement through local conformational remodeling, whereas alterations within structurally constrained regions incur greater functional costs. Together, these findings reveal that region-specific structural constraints govern the evolutionary trajectory of the RBD, with the large loop providing conformational plasticity that permits antigenic variation while preserving essential receptor recognition.

## Results

### Global architecture of JN.4 spike reveals distinct RBD conformational states

Phylogenetic analysis positions JN.4 as a parallel descendant of BA.2.86, diverging from the globally dominant JN.1 lineage through acquisition of the A475V substitution in the RBD and distinct mutations in the N-terminal domain (NTD) (Fig 1A–B). To elucidate the structural consequences of these mutations, we determined the cryo-EM structure of the JN.4 spike ectodomain in complex with human ACE2 at 2.90 Å resolution (S2 Fig, S1 Table).

**Fig 1:**
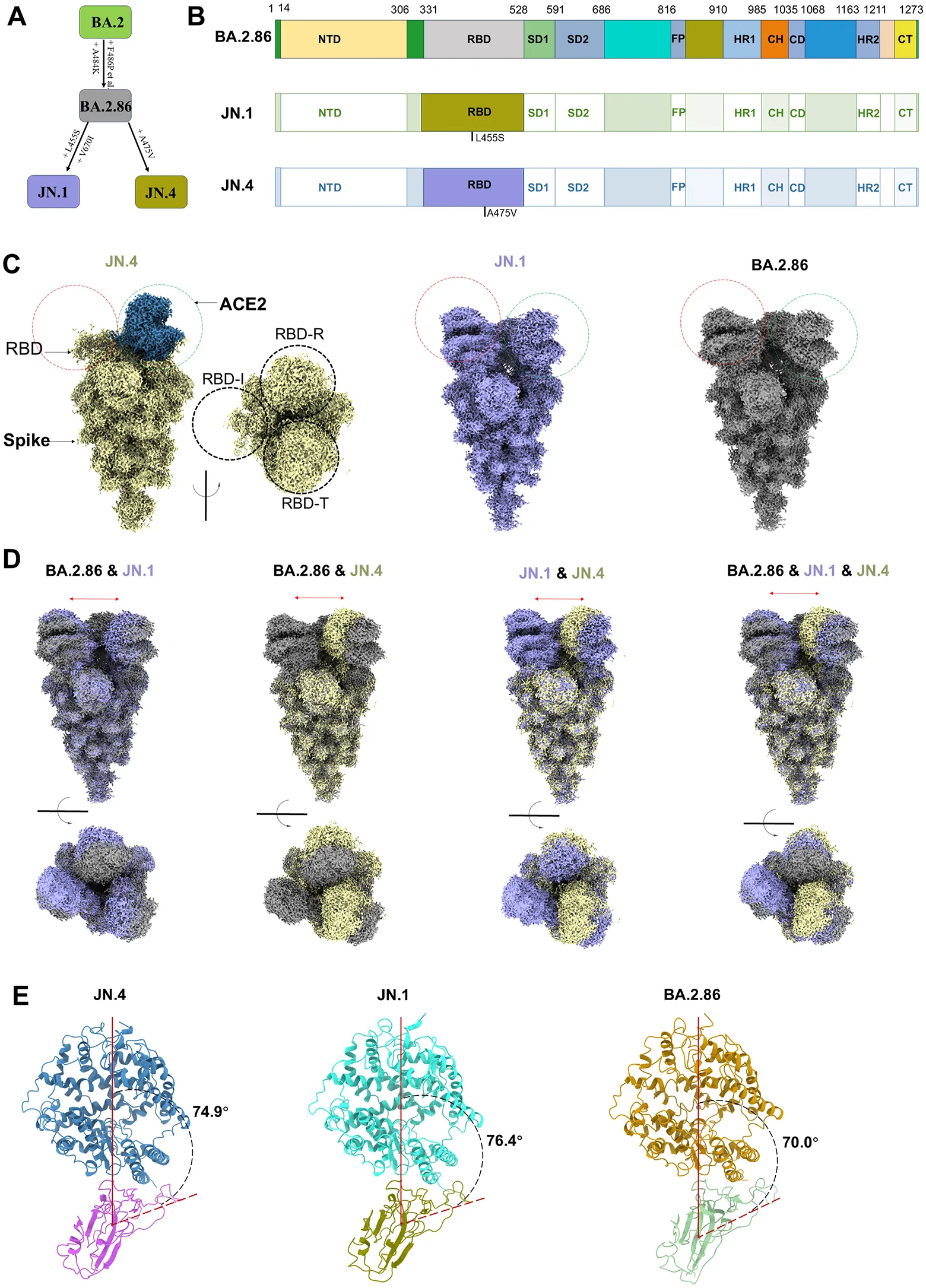
Global structural characterization of the JN.4 spike–ACE2 complex. **(A)** The schematic diagram of SARS-CoV-2 BA.2.86-related subvariants evolution. Some additional mutations in the spike acquired by JN.1 and JN.4 were displayed. Black, purple, and green denote BA.2.86, JN.1, and JN.4, respectively; the same color coding is used in the lower panels. **(B)** Comparison of RBD mutations among BA.2.86, JN.1, and JN.4. JN.4 harbors the A475V substitution in the RBD, distinguishing it from JN.1 harbors the L455S. The ACE2 ectodomain is rendered in steel blue. **(C)** Cryo-EM reconstruction of the ACE2-JN.4 spike ectodomain (Spike-ECD) complex at 2.90 Å resolution. Side and top views show all three RBDs (labeled T, R, I) in the “up” conformation. Red dashed circle highlights RBD-I with reduced ACE2 occupancy; green dashed circle indicates the inward tilt of ACE2 bound to RBD-T. **(D)** Structural alignment of cryo-EM maps of BA.2.86, JN.1, and JN.4. Maps for BA.2.86 (EMD-37928) and JN.1 (EMD-60028) were retrieved from the Electron Microscopy Data Bank. **(E)** Quantification of the deflection angle between the centroid of ACE2 and RBD centroid using RBD residue 481 Cα as reference for BA.2.86, JN.1, and JN.4.

In the global reconstruction, all three RBD protomers (designated T, R, and I in counterclockwise orientation) adopted the “up” conformation compatible with receptor engagement. However, we observed marked asymmetry in ACE2 occupancy: while RBD-T and RBD-R exhibited robust receptor density, RBD-I showed substantially reduced ACE2 binding (Fig 1C, red dashed circle). Strikingly, the ACE2 molecule bound to RBD-T displayed a pronounced inward tilt toward the trimer axis compared to BA.2.86 (Fig 1C, green dashed circle). In addition, among the three ACE2-bound RBD conformations, the RBD-I interface exhibited greater positional deviation relative to RBD-T and RBD-R, adopting a partially open intermediate conformation. This increased conformational heterogeneity may reduce optimal ACE2 accessibility and contribute to the weakened receptor engagement observed for the JN.4 spike (S4 Fig). Cryo-EM map alignment of JN.4 and JN.1 to BA.2.86 reveals a slight global shift in JN.1, with the RBD-T–ACE2 complex tilting toward the trimer center. This inward deflection is more pronounced in JN.4, where the RBD-T likely imposes steric hindrance on RBD-I, partially occluding its position and reducing ACE2 occupancy (Fig 1D).

Quantification of the deflection angle between the ACE2 centroid and RBD centroid revealed an intermediate conformation for JN.4 (74.9°) compared to BA.2.86 (70.0°) and JN.1 (76.4°) (Fig 1E). This structural configuration suggests a potential steric constraint mechanism, in which the inward displacement of RBD-T may partially reduce the spatial accessibility available for neighboring RBDs and thereby influence multivalent ACE2 engagement within the spike trimer. Notably, although A475V and L455S are located within the receptor-binding interface, these substitutions appear to exert distinct structural effects. The loop-proximal A475V substitution is associated with altered conformational organization of the RBD large loop and may influence the broader spike conformational landscape, whereas the L455S substitution located within the central receptor-binding motif primarily affects local receptor-contact interactions.

### Region-specific remodeling mechanisms at the RBD-ACE2 interfaces

To dissect the molecular basis underlying these distinct global conformations, we performed local refinement of the RBD-ACE2 interface, yielding a 3.73 Å density map (Fig 2A, S3 Fig). Structural alignment with JN.1 (PDB: 8ZBQ) and BA.2.86 (PDB: 8WYH) gave RMSDs of 1.1 Å and 1.058 Å, respectively, supporting a conserved binding mode with localized conformational differences. Comparative analysis uncovered divergent remodeling patterns across three distinct interface regions: the anchor region remained structurally invariant, whereas the middle and loop regions underwent mutation-specific reorganization (Fig 2B–C).

**Fig 2:**
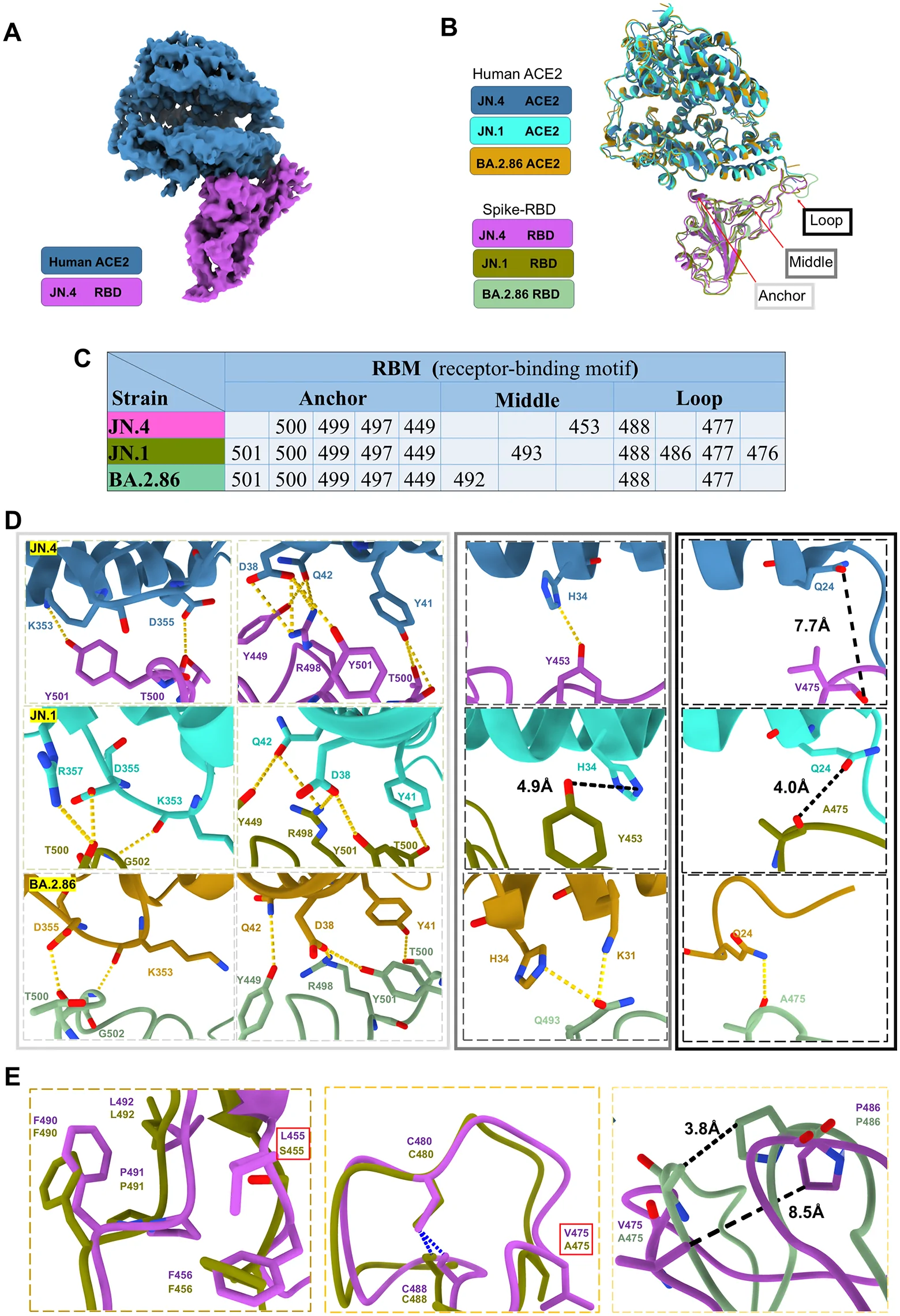
Comparative structural analysis of JN.4, JN.1, and BA. **2.86 RBD–ACE2** interfaces. **(A)** Cryo-EM local refinement of the JN.4 RBD-ACE2 interface. **(B)** Structural alignment with the JN.4, JN.1, and BA.2.86 RBD-ACE2 complex, showing overall conservation of the binding mode and local deviations in three regions. **(C)** Amino acid comparison of the RBM (RBD-ACE2 interface) in BA.2.86, JN.1, and JN.4. **(D)** Analysis of interaction surrounding the same binding site in the RBD-ACE2 complexes of JN.4, JN.1, and BA.2.86**. (E)** Hydrophobic core remodeling. L455S disrupts interactions with Phe456, Phe490, Pro491, and Leu492 in JN.1; A475V induces loop conformational shift, potentially affecting the Cys480–Cys488 disulfide bond.

Within the anchor region, JN.1, JN.4, and BA.2.86 share a highly conserved RBD-ACE2 interaction network, characterized by dense hydrogen bonds and salt bridges that support a similar global binding mode. Specifically, the hydroxyl group of RBD Tyr501 forms hydrogen bonds with ACE2 Lys353 and Asp38 and participates in a local network involving Thr500. Thr500 further interacts with ACE2 Asp355 and Tyr41 through its side chain and backbone carbonyl, respectively. In addition, RBD Arg498 forms three salt bridges with ACE2 Asp38, collectively stabilizing the anchor interface. A subtle variation is observed near Thr500: in JN.4, it interacts with Asp355 and Tyr41, whereas in JN.1, it also forms an additional contact with Arg357, resulting in a slightly expanded hydrogen-bonding network (Fig 2D).

In contrast to the conserved anchor region, BA.2.86, JN.1, and JN.4 exhibit more pronounced divergence in the middle region of the interface. Mutations such as L455S in JN.1 and A475V in JN.4 induce subtle conformational shifts that propagate beyond the local loop and remodel adjacent interactions. In BA.2.86, RBD Gln493 forms hydrogen bonds with ACE2 His34 and Lys31. In JN.1, Ser494 instead interacts with His34, reflecting a reorganization of the local network. In JN.4, the A475V substitution repositions Tyr453 closer to the interface, enabling hydrogen-bond formation with His34, while simultaneously increasing the distance between Ser494 and His34 (~7.5 Å), thereby abolishing their interaction. By comparison, the corresponding distances in JN.1 (~4.9 Å for Tyr453–His34) indicate a weaker interaction. Together, these reciprocal rearrangements illustrate how mutations in adjacent structural regions redistribute local interaction networks while preserving overall binding compatibility (Fig 2D).

Structural analyses reveal that the A475V mutation in JN.4 remodels receptor-binding interactions compared to BA.2.86 and JN.1. While BA.2.86 utilizes a hydrogen bond between the Ala475 backbone and ACE2 Gln24, JN.4 loses this contact due to steric hindrance from the bulkier Val475 side chain. This substitution induces a loop conformational shift that enables an alternative hydrogen bond between RBD Asn477 and ACE2 Ser19, but restricts the interaction network to a single Tyr489–Thr27 contact. Conversely, JN.1 retains multiple hydrogen bonds involving Tyr489, Asn487, Asn477, and Gly476 with ACE2 Tyr83 and Thr20, suggesting a more stable receptor engagement (Fig 2D).

Although the Ala-to-Val substitution represents a chemically conservative change between hydrophobic residues, structural comparisons among BA.2.86, JN.1, and JN.4 indicate a marked and region-specific remodeling of the RBD interface. In particular, the distance between residue 475 and Pro486 increases from 3.8 Å in BA.2.86 to 8.5 Å in JN.4, consistent with a substantial reorganization of loop geometry rather than a simple loss of local packing interactions. This conformational shift is likely to remodel the surrounding hydrogen-bonding network and alter local structural constraints. Mechanistically, JN.1 and JN.4 exhibit distinct effects. In JN.1, the L455S substitution disrupts a hydrophobic interaction network involving Phe456, Phe490, Pro491, and Leu492, leading to partial relaxation of the local hydrophobic core. By contrast, the A475V substitution in JN.4 primarily introduces steric constraints within a polar environment, increasing side-chain bulk and reducing structural accommodation in the loop region. This change may also perturb the geometry of the nearby Cys480-Cys488 disulfide bond, contributing to the observed loop displacement (Fig 2E).

Together, these observations reveal that while the middle-region mutation L455S mainly weakens local hydrophobic packing, the loop-associated mutation A475V exerts a stronger effect on conformational plasticity, driving broader structural rearrangement. This highlights the dominant role of loop flexibility in modulating the ACE2-RBD interaction landscape.

### Evolutionary conservation of hydrophobic anchors within the plastic large loop

As described above, hydrophobic substitutions at residues 455 and 475 within the RBD alter the binding mode between RBD and ACE2 and induce local conformational rearrangements. To further investigate how hydrophobic amino-acid substitutions influence receptor binding and viral infectivity, we systematically compared the RBD sequences of multiple Omicron variants, including XBB.1, XBB.1.5, EG.5.1, BA.2, BA.2.86, JN.1, and JN.4 (Fig 3A).

**Fig 3:**
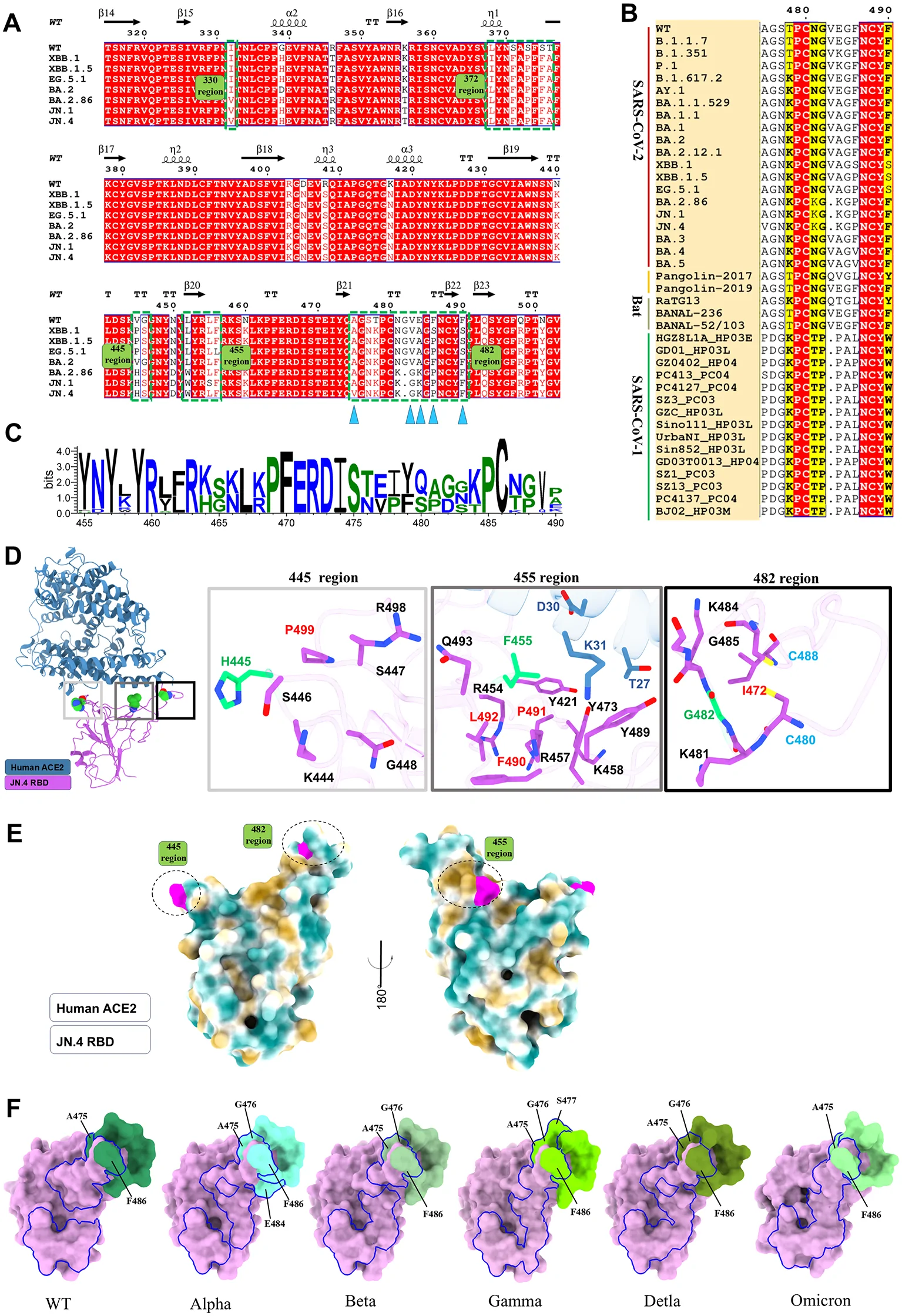
Hydrophobic substitutions highlight evolutionary importance of the large loop. **(A)** Sequence alignment of RBD residues across key Omicron variants (XBB.1, XBB.1.5, EG.5.1, BA.2, BA.2.86, JN.1, JN.4), highlighting the flexible 475–490 loop as a hotspot for hydrophobic substitutions. **(B-C)** Comparative sequence analysis including pangolin- and bat-derived coronaviruses, and SARS-CoV-1, showing recurrent hydrophobic substitutions at residues 475, 483, 484, 486, and 490. Residue numbering follows the SARS-CoV-2 Wuhan (WT) spike protein sequence. **(D)** Comparative analysis of interaction forces surrounding the 445, 455, and 482 regions within the RBD**. (E)** Visualization of the surface hydrophobicity of the JN.4 RBD**. (F)** Structural comparison of the large loop within the RBD across different SARS-CoV-2 variants: WT (PDB: 6M0J), Alpha (PDB: 8DLK), Beta (PDB: 8DLN), Gamma (PDB: 8QLQ), Delta (PDB: 7TEW), and Omicron (PDB: 8AQU).

Sequence alignment revealed that hydrophobic substitutions are enriched in several discrete regions of the RBD, including the 320 region (centered on residue 320), the 372 region, the 445 region (encompassing residues 445–446), the 455 region, and the 482 region (corresponding to ‘the large loop’ characterized in the present study) (Fig 3A, S5 Fig). To further elucidate the evolutionary conservation of these mutation patterns, we expanded our comparative analysis to encompass RBD sequences from diverse sarbecoviruses, spanning pangolin-derived, bat-derived, and SARS-CoV-1-related coronaviruses. Interestingly, hydrophobic substitutions were also frequently observed at analogous positions in these viruses, particularly within the 482 region, suggesting that these sites represent evolutionarily flexible hotspots within the receptor-binding domain (Fig 3A–C, S5 Fig).

Because mutations located directly at the RBD-ACE2 interface are expected to exert the most pronounced structural and functional consequences, we first excluded the 320 and 372 regions, which are spatially distant from the receptor-binding interface. The 445 region, although located on the receptor-binding surface, lies within the relatively rigid “anchor” region where a dense hydrogen-bond network stabilizes ACE2 binding. In addition, the surrounding residues are predominantly polar, with few neighboring hydrophobic residues, suggesting that hydrophobic substitutions in this region are unlikely to substantially alter the overall structural architecture (Fig 3A, D–E).

In contrast, the 455 region contains multiple hydrophobic residues that form a tightly packed hydrophobic cluster. Mutations that replace hydrophobic residues with polar amino acids may weaken these interactions, whereas substitutions among hydrophobic residues with larger side chains may strengthen hydrophobic packing. Because the structural consequences of mutations in this region are largely governed by predictable hydrophobic packing effects, the 455 region was not the primary focus of the present study.

Notably, the 482 region contains a flexible loop spanning residues 475–490, hereafter referred to as the large loop, which represents a recurrent mutational hotspot across SARS-CoV-2 variants. This region frequently accumulates substitutions involving hydrophobic residues. For example, residue 483 is deleted in several variants including BA.2.86, JN.1, and JN.4, while mutations at residue 484 (such as E484A or K484A) are also commonly observed. Despite the absence of a conserved hydrophobic core within this region, recurrent hydrophobic substitutions are frequently detected at positions 475, 483, 484, 486, and 490, often accompanied by distinct conformational rearrangements of the large loop (Fig 3A, D–E).

Despite the lack of conserved hydrophobic residues within this region, hydrophobic substitutions occur recurrently, often leading to distinct structural shifts within the 475–490 loop. Within this region, residues 475, 483, 484, 486, and 490 show particularly frequent hydrophobic substitutions across circulating variants.

To investigate how evolutionary variation within the RBD large loop influences its structural organization, we performed structural alignment across representative SARS-CoV-2 variants, including wild-type (WT) (PDB: 6M0J), Alpha (PDB: 8DLK), Beta (PDB: 8DLN), Gamma (PDB: 8QLQ), Delta (PDB: 7TEW), and Omicron BA.1 (PDB: 8AQU) subvariants. Structural comparison revealed that A475 and F486 represent structurally important residues within the RBD 475–490 large loop. While F486 directly contributes to receptor and antibody recognition through hydrophobic interactions [32,33,38], A475 plays a critical role in maintaining the local conformational landscape of the RBM region. These residues function cooperatively to maintain the overall receptor-binding geometry, effectively acting as hydrophobic anchors that secure ACE2 to RBD. In contrast, the surrounding large loop exhibits substantial conformational variability across variants. Importantly, despite these structural rearrangements, the overall ACE2-RBD interaction remains largely preserved, indicating that loop possesses considerable structural tolerance that allows sequence diversification while maintaining stable receptor recognition (Fig 3F).

Taken together, these observations highlight the large loop as an evolutionarily dynamic structural element within the RBD, where recurrent hydrophobic substitutions reshape the local conformational landscape without substantially disrupting receptor binding. This structural plasticity likely provides a mechanistic basis for how SARS-CoV-2 accommodates mutational diversification while preserving essential receptor-binding functionality.

### Kinetic binding analysis of the SARS-CoV-2 BA.2.86 variant and its specific mutations

To investigate whether hydrophobic substitutions within the large loop alter RBD binding affinity, we introduced nine points mutations into the BA.2.86 subvariant background. To approximate their biological relevance, all selected substitutions correspond to mutations that have been observed in naturally circulating SARS-CoV-2 variants [1,32,39].

Kinetic binding analyses revealed that the equilibrium dissociation constants (K_D_) of BA.2.86, L455S, A475V, del483V, K484A, P486F, P486S, P486V, F490S, and the double mutant L455S+A475V were 20.0 ± 2.2 nM, 29.0 ± 1.8 nM, 69.0 ± 12.0 nM, 11.0 ± 0.8 nM, 39.0 ± 5.7 nM, 38.0 ± 13.0 nM, 230.0 ± 42.0 nM, 91.0 ± 58.0 nM, 200.0 ± 29.0 nM, and 100.0 ± 17.0 nM, respectively (Fig 4). The binding affinity of BA.2.86 was largely consistent with previously reported measurements [32].

**Fig 4:**
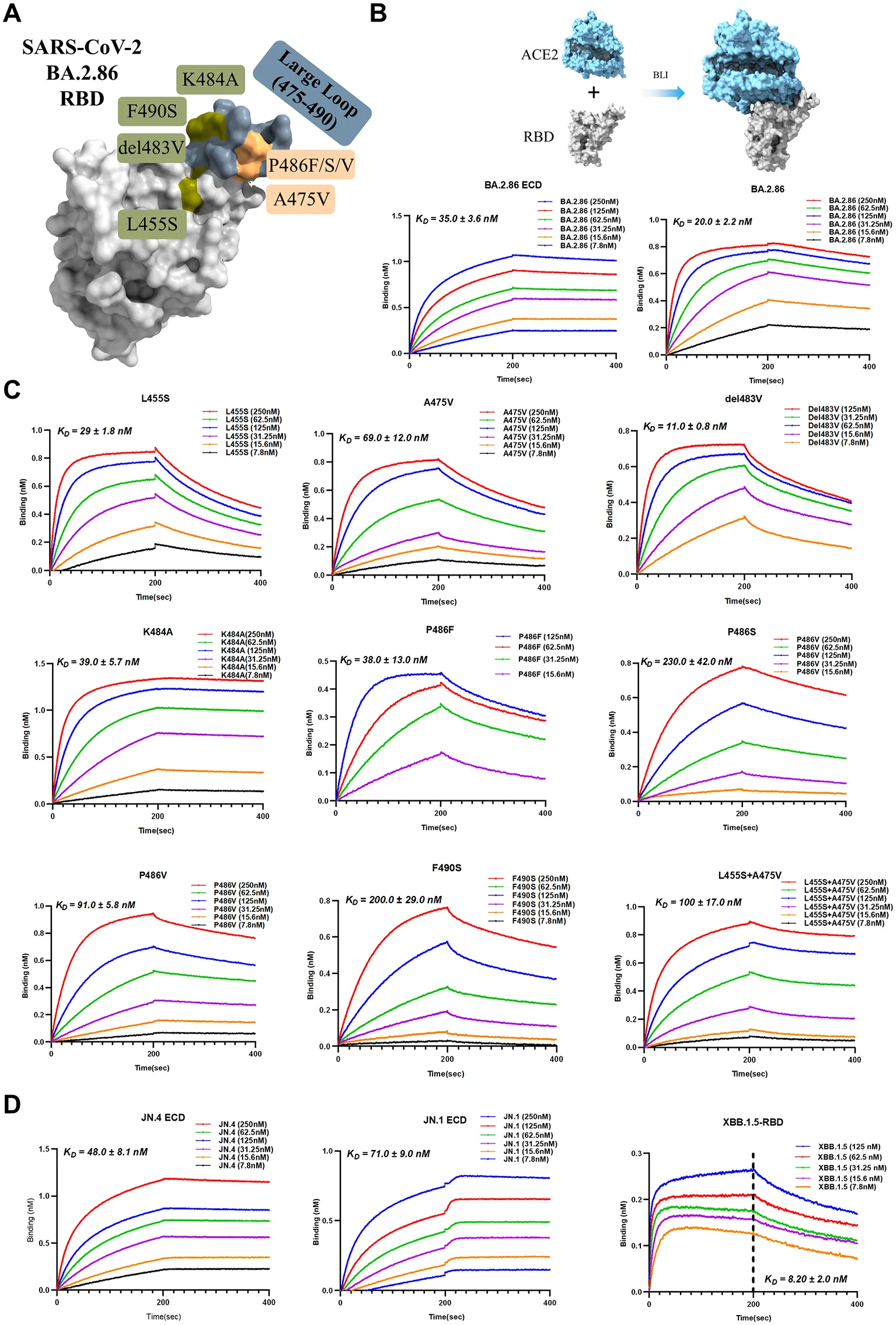
Kinetic binding analysis of BA. **2.86 RBD and point** mutants. **(A)** Structural mapping of hydrophobic residues within the BA.2.86 RBD large loop. **(B-C)** The panels display representative sensor result showing the binding affinity of the BA.2.86 RBD, ECD, and nine specific mutants (L455S, A475V, L455S+A475V, del483V, K484A, P486V, P486F, P486S, F490S) with ACE2-PD. **(D)** Biolayer interferometry analysis of ACE2 binding by full-length spike ectodomains (Spike-ECD) from JN.1 and JN.4 variants and the XBB.1.5 RBD. Colored curves represent graded concentrations of the analyte, as indicated in the panel keys. Equilibrium dissociation constants (K_D_) are presented as mean ± standard deviation (SD) from three independent kinetic experiments. All experiments were performed independently in triplicate (n = 3).

Compared with BA.2.86, both JN.1 and JN.4 exhibited reduced ACE2-binding affinity and decreased thermal stability, suggesting that these substitutions may compromise the stability of the receptor-engaged spike conformation (S6A-B Fig). Notably, BA.2.86 displayed the highest thermal stability among the three variants, whereas JN.4 exhibited the lowest stability, consistent with the structural and functional effects associated with remodeling of the RBD 475–490 large loop.

The JN.1 carrying the L455S substitution exhibited binding affinities comparable to earlier determinations, indicating that the L455S mutation exerts a stronger effect on RBD binding. Restoration of the deleted V483 residue in the BA.2.86 RBD produced only a modest enhancement of ACE2-binding affinity, suggesting that V483 deletion alone does not represent a major determinant of receptor engagement, consistent with prior reports [39]. Similarly, the K484A mutation caused only a slight reduction in binding affinity. Notably, the F490S substitution caused an approximately 10-fold decrease in binding affinity, implying the presence of a hydrophobic interaction network in the vicinity of this residue.

In contrast, the A475V single mutation led to an approximately 3.5-fold reduction in binding affinity relative to BA.2.86, whereas the double mutation L455S+A475V resulted in an approximately 5-fold reduction, indicating a more pronounced effect and suggesting that such a combination may be less likely to arise or persist in circulating variants. Consistent with the structural observations, molecular dynamics simulations further demonstrated that, compared with V475A, the A475V substitution modifies the conformational dynamics of the RBD, including altered flexibility within the 475–490 large loop region (S6 Fig C–D, S2 Table). These findings suggest that A475V reshapes the local structural landscape of the RBD, potentially affecting receptor interaction and antigenic recognition of this region.

Sequence comparison further revealed that the P486 position has undergone substitutions in multiple directions during viral evolution [40,41]. Interestingly, relative to BA.2.86, the binding affinities of the P486S, P486V, and P486F variants decreased sequentially by approximately 11.5-fold, 4.55-fold, and 1.9-fold, respectively. Interestingly, XBB.1.5 showed increased ACE2-binding affinity compared with XBB.1, likely associated with the S486P substitution that remodels the hydrophobic environment within the RBD large loop [38,42]. Conversely, the reduced ACE2-binding affinity of BA.2.86 and JN.1 relative to XBB.1.5 may be related, at least in part, to the S490F difference within this region (Fig 4D). Together, these comparisons demonstrate that hydrophobic remodeling of the RBD large loop represents an important mechanism for fine-tuning ACE2 recognition, with residues 486 and 490 acting as critical modulators of receptor-binding properties.

Together, these results suggest that substitutions within the hydrophobic network may preserve direct chemical interactions while exerting variable effects on RBD–ACE2 engagement depending on their structural context. The functional properties of the RBD 475–490 large loop are therefore likely determined not by individual residue changes alone, but by the coordinated remodeling of multiple structural elements within this flexible region.

### Infectivity and fusogenicity of BA.2.86 and relevant mutations in spike

We first examined the infectivity of pseudoviruses bearing spike of SARS-CoV-2 BA.2.86 and relevant mutations in 293T-ACE2 cell which overexpress human ACE2. As reported previously, BA.2.86 exhibited a similar infectively to WT in 293T-ACE2 cells (1.2-fold increase; *p* > 0.05). BA.2.86 with L455S, del483V, P486F and A475V also did not exhibit a significant change compared with WT (1.1- to 1.2-fold decrease, *p* > 0.05). In contrast, the infectivity of pseudoviruses harboring BA.2.86 with A475V, K484A, P486S, P486V and F490S exhibited a 1.3- to 1.6-fold reduction than WT. Notably, all the mutations exhibited low infectivity compared to BA.2.86 (1.3- to 1.9-fold decrease; *p* < 0.001) (Fig 5A).

**Fig 5:**
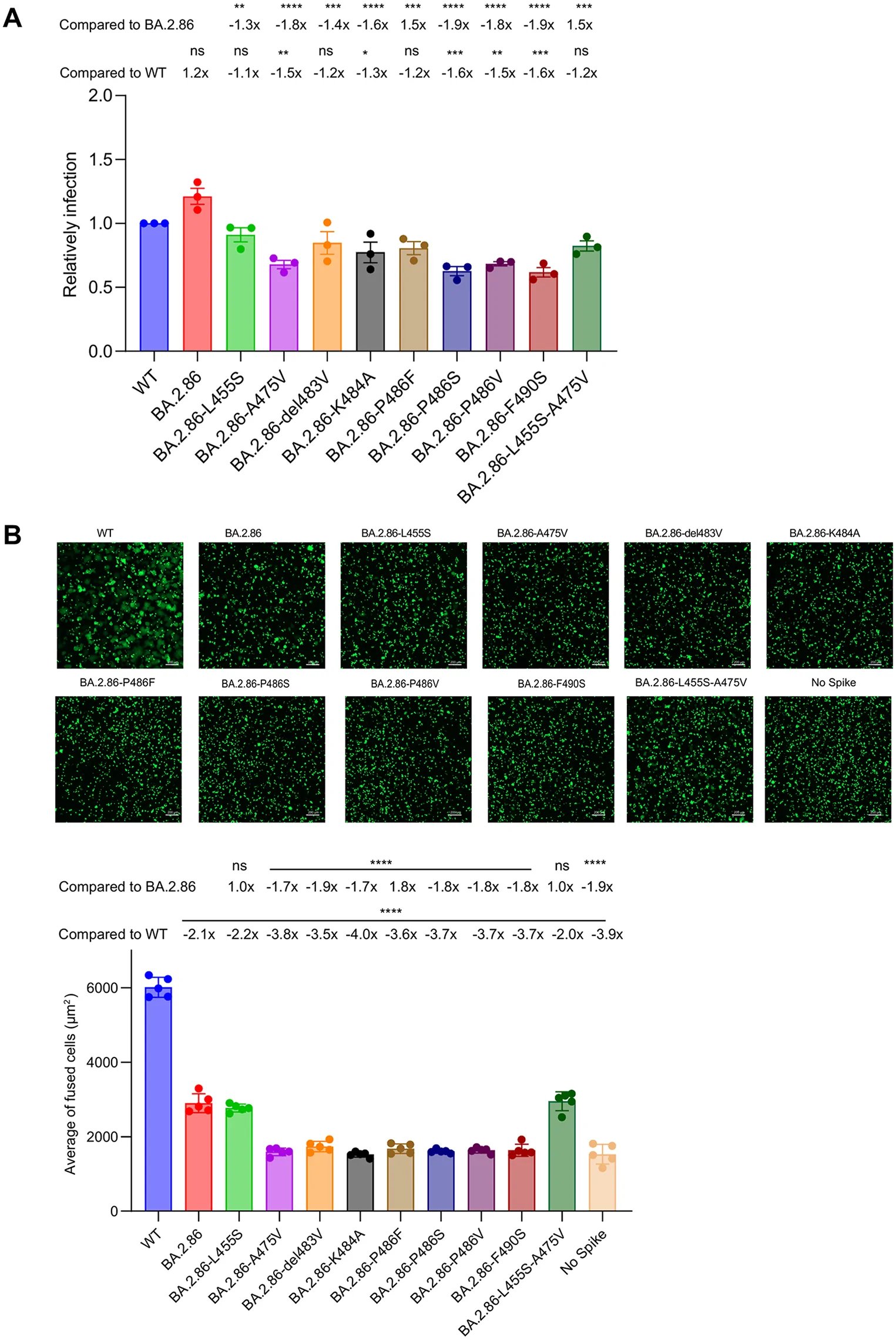
Infectivity and fusogenicity of BA. **2.86 spike and relevant** mutations. **(A)** Infectivity of Omicron BA.2.86 pseudoviruse bearing mutations of interest in spike was determined in 293T-ACE2 cells. Bars represent means ± standard error (SEM) from triplicates. Significance relative to WT spike and BA.2.86 spike was analyzed by a one-way repeated measures ANOVA with Dunnett’s multiple comparisons test (n = 3). p values are displayed as ns *p* > 0.05, **p < 0.01, ***p < 0.001, and ****p < 0.0001. **(B)** HEK-293T cells transfected with plasmid encoding the spike of interest and were cocultured with 293T-ACE2 transfected with eGFP expression vector for 2 h. Cell-cell fusion was imaged and GFP areas of fused cells were quantified. Wild-type spike (wuhan reference strain) and no spike were included as positive and negative control, respectively. Comparisons in extents of cell-cell fusion for each were made against WT spike. Scale bars represent 200 μM. Bars in represent means ± standard error. Dots represent five images from two biological replicates. Statistical significance relative to WT was determined using a one-way repeated measures ANOVA with Dunnett’s multiple comparisons test (n = 5). p values are displayed as ns *p* > 0.05, *p < 0.05, ***p < 0.001, and ****p < 0.0001.

To assess the differences in the ability to mediate membrane fusion of relative mutations in BA.2.86 spike protein, HEK-293T cells transfected with plasmid encoding the spike of interest and were cocultured with 293T-ACE2 transfected with eGFP expression vector for 2 h at 37 ℃. Consistent with the previously studies, the WT spike exhibited significant cell-cell fusion with a relatively weak fluorescence intensity and larger averaged area of the fused cell compared with no spike group. Correspondingly, BA.2.86 and its relevant mutations showed markedly weak cell fusion compared to WT spike. L455S and the L455S/A475V double mutant retained fusion activity comparable to BA.2.86. All other mutants showed minimal activity, similar to the no-spike control. Collectively, these data indicate that substitutions at positions 483–490 further compromise BA.2.86-mediated cell-cell fusion (Fig 5B).

Previously, we characterized the antibody escape profiles of BA.2.86 and JN.1 against a panel of broadly neutralizing antibodies targeting distinct RBD epitopes [32]. Compared with BA.2.86 and JN.1, JN.4 exhibited a largely comparable antibody escape profile, indicating that A475V alone does not substantially enhance resistance to broadly neutralizing antibodies. Similar to the parental BA.2.86 and JN.1 variants, JN.4 showed reduced sensitivity to S2K146 and LY-CoV1404. In contrast, antibodies targeting conserved non-RBM epitopes, including the SA55, C118, S309, and S2H97-like antibodies, retained neutralizing activity against JN.4 (S7 Fig).

These results suggest that the A475V-mediated remodeling of the RBD large loop primarily influences receptor engagement and local conformational dynamics, while exerting limited additional effects on the existing antibody escape landscape of BA.2.86.

## Discussion

SARS-CoV-2 continues to diversify through RBD mutations that reshape receptor engagement and antigenic properties. Within the BA.2.86 sublineage, the contrasting evolutionary trajectories of globally dominant JN.1 and the less successful JN.4 highlight an intriguing paradox, as these closely related variants exhibit substantially different epidemiological outcomes despite extensive sequence similarity. Here, we demonstrate that this divergence stems from region-specific mechanisms of interface remodeling. While JN.1 L455S mutation disrupts hydrophobic packing within the rigid middle region, JN.4 A475V substitution triggers allosteric reorganization of the flexible 475–490 loop, attenuating ACE2 binding avidity and restricting multivalent receptor engagement.

Our structural analysis reveals a bipartite architecture governing RBD-ACE2 interactions. The anchor region (Arg498, Thr500, Tyr501) functions as an invariant structural “rivet,” maintaining a dense hydrogen-bond and salt-bridge network essential for receptor recognition [17,43,44]. This conservation reflects stringent evolutionary constraints imposed by the requirement to preserve host cell entry. In contrast, the adjacent large loop exhibits remarkable structural plasticity, serving as a recurrent mutational hotspot across SARS-CoV-2 variants and related sarbecoviruses [45–50].

Our findings highlight A475 and F486 as structurally important residues within the flexible RBD 475–490 loop. Although substitutions at these positions have emerged during SARS-CoV-2 evolution, their local structural roles remain constrained. F486 functions as a critical hydrophobic interaction hub for receptor and antibody recognition [51,52], whereas A475 contributes to maintaining the conformational organization of the surrounding loop [53]. This finding aligns with studies of influenza hemagglutinin (HA), where hydrophobic substitutions in flexible loops enhance receptor avidity through similar steric effects [54].

Functional analyses further establish the RBD large loop as a structurally plastic yet functionally constrained module. Although hydrophobic substitutions such as A475V can be tolerated through local rearrangement of the receptor-binding interface, they influence ACE2 engagement, spike stability, and viral functional properties, including membrane fusion and infectivity. Because this loop also constitutes an exposed antigenic region of the RBD, such remodeling may reshape the antigenic landscape and modulate neutralizing antibody recognition in an epitope-dependent manner. These observations highlight a fundamental evolutionary balance, where structural plasticity enables adaptation to immune pressure while preserving essential receptor-binding functions. For JN.4, the acquisition of A475V appears to shift this balance, resulting in altered spike properties that may constrain its evolutionary advantage compared with other Omicron descendants.

Collectively, our findings reveal the RBD 475–490 large loop as a structurally flexible yet functionally constrained element that contributes to SARS-CoV-2 adaptation. Although this region undergoes recurrent sequence variation during viral evolution, key hydrophobic residues, particularly A475 and F486, are functionally constrained to preserve the receptor-binding framework. This balance between structural plasticity and functional stability enables SARS-CoV-2 to explore antigenic variation while maintaining essential receptor recognition, but also imposes evolutionary trade-offs that shape the fitness landscape of emerging variants.

## Materials and methods

### Protein expression and purification of trimeric S proteins and RBDs

The extracellular domain (ECD) (1–1208 a.a) of S protein of SARS-CoV-2 Omicron variant BA.2.86, JN.1, and JN.4 were cloned into the pCAG vector (Invitrogen) with six proline substitutions at residues 817, 892, 899, 942, 986, and 987 and a C-terminal T4 fibritin trimerization motif followed by Flag and 10 × His tag, respectively. A “GSAS” mutation at residues 682–685 was introduced into ECD to prevent the host furin protease digestion. These constructs were hereafter referred to as WT-S, XBB.1.5-S, and BA.2.86-S.

The RBD (319–541 a.a) of S protein from SARS-CoV-2 WT strain and Omicron variant BA.2.86, JN.1, and JN.4 were cloned into the pCAG vector (Invitrogen) with an N-terminal signal peptide of secreted luciferase and a C-terminal 6 × His tag, respectively. These residues numbers mentioned above are that relative to the spike (WT). The PD (19–615 a.a) of human ACE2 was also cloned into the pCAG vector (Invitrogen) with an N-terminal signal peptide of secreted luciferase and a C-terminal Flag tag.

The recombinant protein was overexpressed using the HEK293F mammalian cells at 37 °C under 5% CO_2_ in a Multitron-Pro shaker (Infors, 130 rpm). When the cell density reached 2.0  ×  10^6^ cells/mL, the plasmid was transiently transfected into the cells. To transfect one liter of cell culture, about 1.5 mg of the plasmid was premixed with 3 mg of PEIs in 50 mL of fresh medium for 15 mins before adding to cell culture. Cells or medium was collected by centrifugation at 4000  ×  *g* for 15 min after 60 h transfection.

The secreted ECD and RBD of S protein were purified by Ni-NTA affinity resin (Qiagen). The nickel resin loaded was rinsed with the wash buffer 1 containing 25 mM HEPES (pH 7.5), 150 mM NaCl and washed with wash buffer 2 containing 25 mM HEPES (pH 7.5), 150 mM NaCl and 30 mM imidazole. Protein was eluted by wash buffer 2 plus 300 mM imidazole. Then the Ni-NTA eluent of ECD was subjected to size-exclusion chromatography (Superose 6 Increase 10/300 GL, GE Healthcare) in buffer containing 25 mM HEPES (pH 7.5), 150 mM NaCl. The peak fractions were collected and stored at −80 °C. The Ni-NTA eluent of RBD was subjected to size-exclusion chromatography (Superose 6 Increase 10/300 GL, GE Healthcare) in buffer containing 25 mM HEPES (pH 7.5), 150 mM NaCl. The factions were collected for structural analysis and measurement of RBD binding to human ACE2-PD by BLI.

The secreted PD was purified by anti-FLAG M2 affinity resin (Sigma Aldrich). After loading two times, the anti-FLAG M2 resin was washed with the wash buffer 3 containing 25 mM HEPES (pH 7.5), 150 mM NaCl. The protein was eluted with the wash buffer 3 plus 0.2 mg/mL flag peptide. The eluent of PD was then concentrated and subjected to size-exclusion chromatography (Superose 6 Increase 10/300 GL, GE Healthcare) in buffer containing 25 mM HEPES (pH 7.5), 150 mM NaCl.

The JN.4-S was incubated with IgG/PD at a molar ratio of about 1:1.2 for 1 h. To remove excessive IgG/PD, the mixture was subjected to size-exclusion chromatography (Superose 6 Increase 10/300 GL, GE Healthcare) in buffer containing 25 mM HEPES (pH 7.5), 150 mM NaCl. The peak fractions containing protein complex were collected for EM analysis.

### Measurement of Omicron subvariants and WT strain RBD binding to human ACE2-PD by biolayer interferometry

The binding between PD and RBD of WT and other subvariants were performed using Octet Red96e (ForteBio). The PD of ACE2 was biotinylated using Biotinylation Kit (Genemore, 1828M) and loaded to octet SA biosensor (Sartorius). The association and dissociation of PD-coated biosensors with different concentrations of RBD of SARS-CoV-2 S protein were recorded in binding buffer (25 mM HEPES, 150 mM NaCl pH 7.5, 0.04% Tween-20). Data were analyzed by Octet Data Analysis HT 12.0 software. The reference sample and reference sensor were subtracted, and *K*_D_ values were analyzed using a 1:1 global fit model. Data were plotted using Prism V8.0 software (GraphPad).

### Cryo-EM sample preparation and data acquisition

The JN.4-spike-ECD-PD complex was concentrated to ~3 mg/mL applied to two 400-gold-mesh cryo-EM grids. Aliquots (3.5 μL) of the protein were placed on glow-discharged holey carbon grids (Quantifoil Au R1.2/1.3). The grids were blotted for 3.0 s and flash-frozen in liquid ethane cooled by liquid nitrogen with FEI Mark II Vitrobot at 6 °C and 100% humidity. The prepared grids were transferred to a Titan Krios operating at 300 kV equipped with Gatan K3 detector and GIF Quantum energy filter. Movie stacks were automatically collected using AutoEMation [55] and EPU software, with a slit width of 20 eV on the energy filter and a defocus range from -1.0 µm to -1.8 µm in super-resolution mode at a nominal magnification of 105,000 × . Each stack was exposed for 1.14 s with an exposure time of 0.035 s per frame, resulting in a total of 32 frames per stack. The total dose rate was approximately 50 e^-^/Å^2^ for each stack. The stacks were motion corrected with MotionCor2 [56] and binned 2-fold, resulting in a pixel size of 0.855 or 0.83 Å/pixel. Meanwhile, dose weighting was performed [57]. The defocus values were estimated with Gctf [58].

### Data processing

Particles for JN.4-spike-ECD-ACE2 were automatically picked using cryoSPARC from manually selected micrographs. After 2D classification with cryoSPARC, good particles were selected and subject to multiple cycle of heterogeneous refinement without symmetry using cryoSPARC [59]. The good particles were selected and subjected to Local CTF Refinement, Non-uniform Refinement [60], resulting in the 3D reconstruction for the whole structures. For all datasets, symmetry was not applied until final refinement stages. For the ECD-PD complex, *C1* symmetry were applied, respectively, followed by symmetry expansion of the particles and local refinement for one asymmetric unit. The resolution was estimated with the gold-standard Fourier shell correlation 0.143 criterion [61] with high-resolution noise substitution [62]. Refer to S2 Figs and S1 Table for details of data collection and processing.

### Model building and structure refinement

Maps were sharpened using phenix.auto_sharpen with half-maps (v.1.21). For model building of the complex of ECD-PD, the atomic model of the ECD-PD (PDB: 8WYH) were used as templates, which were fitted into many whole cryo-EM maps of the complex with the UCSF Chimera package [63] (http://www.rbvi.ucsf.edu/chimera), respectively. Each residue was manually checked with the chemical properties taken into consideration during model building by COOT [64]. Several segments, whose corresponding densities were invisible, were not modeled. The completed model of ECD-PD complex was finally refined using PHENIX [65] with secondary structure and geometry restraints to prevent overfitting. To monitor the potential overfitting, the model was refined against one of the two independent half maps from the gold-standard 3D refinement approach. The stereochemical quality of the model was assessed with MolProbity [66]. The structures were visualized, and figures were rendered using either ChimeraX [67] or PyMOL (https://pymol.org/). Data collection, 3D reconstruction, and model-building statistics are summarized in S1 Table.

### Thermal Shift Assay (TSA)

Thermal stability of six SARS-CoV-2 Spike protein constructs (BA.2.86, JN.1, and JN.4 variants, each as full-length ECD with HexaPro 6P stabilizing mutations and as isolated RBD) was assessed by Thermal Shift Assay using SYPRO Orange on a real-time PCR instrument (Roche LightCycler 480). Samples were heated from 37℃ to 95℃in ~1℃steps (63 data points per well), with three technical replicates per construct. Relative fluorescence units (RFU) were recorded at each temperature to generate melt curves. Melt curves were fit by nonlinear least-squares regression using ‘scipy.optimize.curve_fit’ (Python 3, SciPy) with bounded parameter optimization. Spike ECD curves, which display multiple unfolding transitions, were fit to a global multi-sigmoid model comprising a linear baseline, three Boltzmann sigmoid terms, and a high-temperature fluorescence decay term. Two melting temperatures were reported: Tm1 (global ECD unfolding, ~ 63–65 °C) and Tm2 (S2 core unfolding, ~ 85–86 °C), each taken as the midpoint of the corresponding sigmoid. A shallow RFU minimum at ~54 °C was confirmed as the intersection of the declining baseline with the first unfolding onset (linear fit R² > 0.99, 37–52 °C) and was not reported as an independent Tm. RBD curves, showing a single major transition, were fit to a single Boltzmann sigmoid with a decay term; Tm was taken as the sigmoid midpoint (~58–60 °C). All 18 fits achieved R² ≥ 0.994. Tm reliability was confirmed by derivative cross-validation (first-derivative peak vs. sigmoid midpoint, ΔTm < 0.5 °C) and bootstrap 95% confidence intervals (N = 500 resampling; all CI widths < 1.3 °C). All analyses were performed in Python 3 (NumPy, SciPy, pandas, matplotlib).

### Molecular Dynamics (MD)

Before building the system for MD simulation,the V475A structure was obtained from the A475V structure, using the chimerax software after which the CHARMM-GUI web interface [68], was used to build molecular dynamics (MD) simulation systems for both A475V and V475A. All ionisable residues were protonated at pH 7.0. A cubic explicit water box was added to each system to mimic physiological ionic strength of 150 mM NaCl, and the TIP3P water model was used in these simulations. All MD simulations were carried out with GROMACS and the CHARMM36 force field was used for the systems. To remove any undesirable contacts between atoms, each system was first minimized for 5,000 steps, using both steepest descent and conjugate gradient methods, before equilibration. The equilibration was then performed for 2ns under NPT ensemble at 1.0 bar and at 303 K. After equilibration a production MD simulation of 300 ns was performed for each system.The free binding energy for both systems was calculated using the Molecular Mechanics/Generalized Born Surface Area (MM/GBSA) approach with Generalized Born OBC model using a salt concentration of 150 mM, as described by Wu et al., (2022) [69]. Snapshots extracted from the final 100ns of the trajectory were used for the MMGBSA analysis.

### Plasmid

The Codon-optimized spike gene of SARS-CoV-2 Wuhan reference strain (WT) and BA.2.86 variant lacking C-terminal 19 amino acids were cloned into pVAX1 expression vector (Spike-Δc19) as previously described [32].The expression vectors of spike with L455S, A475V, del483A, K484A, P486F, P486S, P486V, F490S and L455S/A475V mutations were constructed by point mutation based on the BA.2.86 spike using the Mut Express II Fast Mutagenesis Kit V2 according to the manufacturer’s instructions (Vazyme Biotech).

### The generation and infectivity of pseudoviruses

SARS-CoV-2 pseudoviruses were produced by co-transfecting Spike-Δc19 and pNL4–3-Luc-R-E- into HEK293T cells in a 1:2 ratio using EZ transfection reagent according to the manufacturer’s instructions (Life-iLab). The culture supernatants were collected at 48 h post-transfection and clarified by centrifugation and stored at −80 °C for subsequent assays. The generation of pseudoviruses harboring BA.2.86 and related mutations was determined by measuring the luciferase activity in the HEK293T-ACE2 cells using Bright-Lite Luciferase reagent (Vazyme Biotech). The quantification of pseudoviruses was confirmed using the human immunodeficiency virus type 1 (HIV-1) p24 / capsid protein p24 Elisa Kit according to the manufacturer’s instructions (Sino Biological).

To measure the infectivity of pseudoviruses, serial twofold dilution of the equal amounts of the quantified pseudoviruses were prepared in a 96-well plate using culture medium and incubated at 37 ℃ for 30 min. And then the serially diluted pseudoviruses were then added into the HEK-293T-hACE2 cells at 2.5 × 10^4^ per well in 96 white cell wells and incubated for 48 h at 37 ℃, 5% CO_2_. The infectivity of pseudoviruses was determined by measuring the luciferase activity in the 293T-ACE2 cells and compared with WT and BA.2.86.

### Cell-cell fusion

To compare differences of fusogenicity of the spikes, 293T cells were transfected with plasmid encoding the spike of interest as the effector cells (293T-Spike) and 293T-ACE2 cells were transfected with eGFP expression vector as target cell (293T-ACE2-eGFP), respectively. After transfection for 24 hours, target 293T-ACE2-eGFP cells were seeded at a density of 7.5 × 10^5^ into 6-well plates. And then, effector 293T-Spike cells were digested by trypsin and added into target 293T-ACE2-eGFP cells at a density of 1.2 × 10^6^ and co-incubated at 37 ℃ for 2 hours. Five fields in each well were randomly selected and imaged using a BioTek Cytation 7 imaging reader and the Gen 5 imager software was used to calculate the fused and unfused cells and the area of the fused cell based on the GFP signal [33]. The fused cells are at least twice as large as the unfused cells, and the fluorescence intensity in the fused cell became weak compared with the unfused cells [70]. Scale bars represent 200 μm and one representative image was selected for presentation.

### Sequencing of SARS-CoV-2 phylogenetic analyses

A total of 52 representative SARS-CoV-2 Spike amino acid sequences were collected from the GISAID and NCBI databases. The Spike sequence of the Wuhan-Hu-1 reference strain was used as the prototype reference for residue numbering and sequence comparison. Multiple sequence alignment was performed using ClustalW [71]. Phylogenetic relationships were reconstructed using the Neighbor-Joining method implemented in MEGA11 [72,73]. Evolutionary distances were estimated using the Jones–Taylor–Thornton (JTT) amino acid substitution model, and the tree was constructed based on pairwise deletion of gaps and missing data [74]. Statistical support for the inferred phylogenetic relationships was evaluated using 1,000 bootstrap replicates [75]. Phylogenetic trees were subsequently visualized and annotated using FigTree v1.4.4 and the Interactive Tree of Life (iTOL) platform. All evolutionary analyses were performed in MEGA11.

### Statistical analyses

Differences between experimental groups were determined by using an unpaired t-test and analysis of variance (ANOVA) in the GraphPad Prism software (GraphPad Software Inc.).

## Supporting information

S1 Fig The relative frequencies of SARS-CoV-2 variants over time. (A) The relative frequencies of SARS-CoV-2 variants over time. The relative frequencies of SARS-CoV-2 variants over time. The data to produce the chart were collected from the GISAID database and updated on 19 July 2026. Of which, XFG* combine its main sublineages including XFG.6, XFG.5.1, XFG.4, XFG.3.4.1, XFG.3.4, XFG.3, XFG.2, XFG.14.1 and XFG.1.1variants. XBB* combine its main subvariants including XBB.2.3, XBB.1.9, XBB.1.5, XBB.1.22, XBB.1.16.6, XBB.1.16.1, XBB.1.16 and XBB.1 variants. KP* include the KP.1.1, KP.2, KP.2.3, KP.3, KP.3.1, KP.3.1, KP.3.2 and KP.3.3 sublineages. JN.1*combine the JN.1 and its main subvariants including JN.1.1, JN.1.1.1, JN.1.13.1, JN.1.16, JN.1.16.1, JN.1.18.5, JN.4, JN.4.5 and JN.1.7 variants. And the emerging PQ* include the PQ.43, PQ.2.8.1, PQ.2.1, PQ.2 and PQ.16.1.1 variants. (B) Phylogenetic analysis of circulating SARS-CoV-2 strains. Phylogenetic tree of SARS-CoV-2 Spike protein, with variants of concern highlighted in bold black. (C) Overall display of prevalence of subvariant with A475V in spike. The sequence number of main SARS-CoV-2 variants with A475V collected in the GISAID for temporal distributions and proportion.
(TIFF)

S2 Fig Flowchart of JN.4-ACE2 for cryo-EM data processing. Representative cryo-EM micrograph of JN.4-ACE2 complex particles. Flowchart of the cryo-EM data processing. Starting with 2151 micrographs, particles are selected through motion correction and template picking, resulting in 815,693 particles. After 2D classification and ab-initio reconstruction, 297,871 good particles are used for Non-uniform Refinement. Various refinement processes follow, leading to final 3D maps at 2.90 resolution. Key steps in the process are highlighted in red.
(TIFF)

S3 Fig Cryo-EM analysis of S-ECD from JN.4 with ACE2. (A) Local resolution map for the 3D reconstruction of the overall high resolution structure. Euler angle distribution in the final 3D reconstruction of overall map. FSC curve of the refined model of S-ECD (JN.4)-ACE2 versus the overall structure that it is refined against. (B) Local resolution map for the 3D reconstruction of the overall first RBD with ACE2 structure. Euler angle distribution in the final 3D reconstruction of overall map. FSC curve of the refined model of RBD (JN.4)-ACE2 versus the overall structure that it is refined against.
(TIFF)

S4 Fig Structural comparison of JN.4 and BA. 2.86 spike–ACE2 complexes. (A) Superposition of full-length JN.4 and BA.2.86 spike trimer–ACE2 complexes. The comparison highlights conformational differences across the three protomers within each trimer: RBD-T, RBD-R, and RBD-I. Structural alignment of corresponding protomers between the two variants reveals mutation-dependent deviations in RBD orientation and loop geometry. (B) Structural alignment of the two RBD–ACE2 interfaces within the JN.4 trimer. (C) Modeling of the ACE2 dimer (PDB: 6M17) onto the JN.4 full-length spike trimer. The physiological dimeric state of ACE2 was superimposed onto the cryo-EM density to evaluate potential steric clashes and geometric constraints during multivalent receptor engagement.
(TIFF)

S5 Fig Sequence alignment of RBD in circulating SARS-CoV-2 strains. The RBD sequence alignment in epidemic SARS-CoV-2 isolates. 482 region (blue 190 dashed lines).
(TIFF)

S6 Fig Thermal stability and molecular dynamics analyses of SARS-CoV-2 Omicron variant spike proteins and receptor-binding domains. (A–B) Thermal shift assays assessing the conformational stability of full-length spike (S) trimers (A) and isolated RBD (B) from JN.1, JN.4, and BA.2.86 variants. Normalized relative fluorescence units (RFU, 0–1) reflect the extent of SYPRO Orange dye binding to solvent-exposed hydrophobic regions upon thermal denaturation. The melting temperature (Tm), defined as the inflection point of the sigmoidal denaturation curve, serves as a quantitative metric of global protein stability; higher Tm values indicate greater thermal stability. (C) Root-mean-square deviation (RMSD) of Cα atoms for the RBD–ACE2 complex during 300-ns molecular dynamics simulations. The V475A exhibits lower RMSD compared to the A475V, indicating reduced conformational fluctuation and increased structural rigidity of the complex. (D) Per-residue root-mean-square fluctuation (RMSF) of the RBD large loop. A475V shows attenuated flexibility relative to V475A, particularly around residues 475–490, consistent with steric constriction imposed by the valine side chain.
(TIFF)

S7 Fig Antigenic landscape of the RBD large loop and neutralization profile of JN.4 pseudoviruses against antibodies. (A) Structural mapping of antigenic epitopes from eight distinct antibody classes onto the SARS-CoV-2 RBD large loop (residues 475–490). The epitopes are color-coded by antibody class and overlaid on the JN.4 RBD structure: Class 1 (B38, PDB: 7BZ5), Class 1/2 (BD23, PDB: 7BYR), Class 2 (LY-CoV555, PDB: 7KMG), Class 2/3 (REGN10987, PDB: 6XDG), Class 1/4 (C118, PDB: 7RKV), Class 3 (S309, PDB: 7RKU), Class 4 (EY6A, PDB: 6ZCZ), and Class 5 (S2H97, PDB: 7M7W). Key contact residues within the 475–490 loop are highlighted as spheres, with hydrophobic positions (A475, F486) annotated to illustrate their proximity to distinct paratopes. (B)The neutralization of mAbs against JN.4 pseudoviruses. The 50% inhibitory concentration (IC_50_) values are means of two independent experiments. The neutralization potency is marked in the different color. Red: high, yellow: moderate, green: weak, gray: non-neutralization (IC_50_ > 50μg/mL). The neutralization breadth is deﬁned as the percentage of pseudoviruses neutralized by each mAb. Theneutralization potency is calculated by the geometric mean of neutralizing values <50μg/mL.The neutralization curves of one out of two independent experiments with similar results were showed and a cut-off value of 50% in neutralization was indicated by the dotted horizontal line. All mAbs were serially 5-fold diluted from the highest concentration of 50 ug/mL to a total of 8 dilutions.
(TIFF)

S1 Table Data collection, refinement and validation statistics. (DOCX)

S2 Table The contributions of various energies to the binding energy. (DOCX)
