# Supplementary material for: Hydrophobic remodeling of the SARS-CoV-2 RBD large loop attenuates ACE2 engagement and viral fitness
Source: PLoS Pathog. 2026 Oct 26;22(10):e1014742. doi: 10.1371/journal.ppat.1014742 (PMC13722295; doi:10.1371/journal.ppat.1014742)
Supplement: Supplementary file 1 [file ppat.1014742.s008.docx]

**Table S1 Data collection, refinement and validation statistics.**

| **Data collection** | | |
| --- | --- | --- |
| EM equipment | Titan Krios | |
| Voltage (kV) | 300 | |
| Detector | Gatan K3 Summit | |
| Energy filter | Gatan GIF Quantum | |
| Pixel size (Å) | 0.855 | |
| Electron dose (e-/Å2) | 50 | |
| Defocus range (μm) | -1.2~ -1.8 | |
| Sample | The global of JN.4-ECD-ACE2 | The local of JN.4-RBD-ACE2 |
| Number of collected micrographs | 2151 | |
| **3D Reconstruction** |  |  |
| Software | cryoSPARC | cryoSPARC |
| Sample | Overall | Overall |
| Number of used particles | 297,871 | 297,871 |
| Resolution (Å) | 2.90 | 3.73 |
| Symmetry | C1 | C1 |
| Map sharpening B-factor (Å2) | -100.2 | -177.3 |
| **Refinement** |  |  |
| Software | cryoSPARC | cryoSPARC |
| Protein residues | 5415 | 184 |
| Side chains assigned | 5415 | 184 |
| R.m.s deviations |  |  |
| Bonds length (Å) | 0.008 | 0.07 |
| Bonds Angle (˚) | 0.878 | 1.221 |
| **Validation** |  |  |
| Clash score | 8.62 | 7.71 |
| rotamer outliers (%) | 0.08 | 1.06 |
| Ramachandran plot statistics (%) |  |  |
| Favored | 95.18 | 87.68 |
| Allowed | 5.7 | 5.26 |
| Outlier | 0.54 | 1.06 |
