# Supplementary material for: Hydrophobic remodeling of the SARS-CoV-2 RBD large loop attenuates ACE2 engagement and viral fitness
Source: PLoS Pathog. 2026 Oct 26;22(10):e1014742. doi: 10.1371/journal.ppat.1014742 (PMC13722295; doi:10.1371/journal.ppat.1014742)
Supplement: Supplementary file 2 [file ppat.1014742.s009.docx]

**Table S2 The contributions of various energies to the binding energy**

| systems | ΔVDWAALS | ΔEEL | ΔEGB | ΔESURF | ΔGGAS | ΔGSOLV | ΔTOTAL |
| --- | --- | --- | --- | --- | --- | --- | --- |
| A475V | -77.70 ± 4.57 | -1272.69 ± 48.19 | 1335.83 ± 47.11 | -10.28 ± 0.67 | -1350.39 ± 48.84 | 1325.55 ± 47.02 | -24.84 ± 6.21 |
| V475A | -90.53 ± 6.54 | -1335.66 ± 51.46 | 1403.11 ± 49.57 | -12.16 ± 0.84 | -1426.31 ± 52.19 | 1390.96 ± 49.24 | -35.24 ± 7.15 |
